# Latest Advances in Regional Anaesthesia

**DOI:** 10.3390/medicina60050735

**Published:** 2024-04-28

**Authors:** Frances Fallon, Aneurin Moorthy, Conor Skerritt, Gillian G. Crowe, Donal J. Buggy

**Affiliations:** 1 Department of Anaesthesia, Mater Misericordiae University Hospital, Eccles St, D07 WKW8 Dublin, Ireland; ffallon@tcd.ie; 2 Department of Anaesthesia, National Orthopaedic Hospital Cappagh/Mater Misericordiae University Hospital, Eccles St, D07 WKW8 Dublin, Ireland; aneurin.moorthy@gmail.com (A.M.);; 3 School of Medicine, University College Dublin, D04 V1W8 Dublin, Ireland; 4 Department of Anaesthesia, Cork University Hospital, Wilton, T12 DC4A Cork, Ireland; 5 The ESA-IC Oncoanaesthesiology Research Group and Outcomes Research, Cleveland, OH 44195, USA

**Keywords:** regional anaesthesia, acute pain, surgery, anaesthesia

## Abstract

Training and expertise in regional anaesthesia have increased significantly in tandem with increased interest over the past two decades. This review outlines the most recent advances in regional anaesthesia and focuses on novel areas of interest including fascial plane blocks. Pharmacological advances in the form of the prolongation of drug duration with liposomal bupivacaine are considered. Neuromodulation in the context of regional anaesthesia is outlined as a potential future direction. The growing use of regional anaesthesia outside of the theatre environment and current thinking on managing the rebound plane after regional block regression are also discussed. Recent relevant evidence is summarised, unanswered questions are outlined, and priorities for ongoing investigation are suggested.

## 1. Introduction

Regional anaesthesia is a subspecialty of anaesthesia which has undergone a renaissance in recent years. Regional anaesthesia is the application of transiently nerve-inhibiting drugs, usually local anaesthetic (LA), to an individual nerve, plexus of nerves, or anatomical plane through which nerves pass, in order to render a distal site (away from the needle site) anaesthetised. It can be used for surgical anaesthesia or analgesia, especially postoperatively. Over the last two decades, a growing appreciation for the application of regional techniques has evolved within the anaesthesia community. This is echoed in the literature with original investigations and new innovations. Since the introduction of ultrasound technology, regional anaesthesia has become more efficacious, safer, and more accessible to anaesthetists in many different fields of work [1]. It is often incorporated as part of a multimodal approach to both anaesthesia and analgesia, with much of its popularity related to its opioid-sparing effects [2]. Its use, particularly in trauma-related injuries, has gained a lot of momentum in recent years. This has seen its application extended to both surgical and non-surgical patients with encouraging data regarding analgesic effects and outcomes for patients [3]. Such is the appreciation for regional anaesthesia, that it has also featured in a number of surgical Enhanced Recovery After Surgery (ERAS) protocols, contributing to reduced recovery times, lengths of stay, and morbidities [4,5]. A trend has emerged in favour of motor-sparing blocks for lower limb surgeries in recent years, and for the role of regional anaesthesia in patients with significant comorbidities undergoing surgery [6,7]. This article will consider some recent advances in regional anaesthesia, including fascial plane blocks, local anaesthetic pharmacology, neuromodulation for acute pain, and regional anaesthesia in non-surgical patients. Finally, we will consider the potential future course of regional anaesthesia and highlight some questions that remain unanswered.

## 2. Fascial Plane Blocks

The fascial plane has become an anatomical target for regional anaesthesia over the past decade [8]. Fascial plane blocks are characteristically large-volume blocks that target musculofascial planes through which different nerves pass, as opposed to traditional distinct nerve targets. An overall consensus on how these blocks work has yet to be established. It is hypothesised that a number of factors contribute to the mechanism of analgesia: the blockade of both sensory afferent nerves travelling within the fascia and nociceptors in nearby tissue, the systemic absorption of LA, and the inhibition of sympathetic nerves travelling within the fascial plane may have a role [9,10]. The trajectory of these different nerves through fascial planes varies considerably, making the predictability of blockade a challenge [11]. Further adding to the unpredictability of fascial plane blocks is the wide variability of fascia itself. Generally, the effectiveness of a fascial plane block is thought to be influenced by its spread. This spread relies on the anatomical structure of the fascia which is not synonymous across all patients. The composition of fascia is affected by ageing, trauma, and conditions such as diabetes mellitus. Lines of fusion can form secondary to adhesion formation and these too have been theorised as a hindrance to the spread of LA, resulting in an unpredictable block [11].

**Figure 1:**
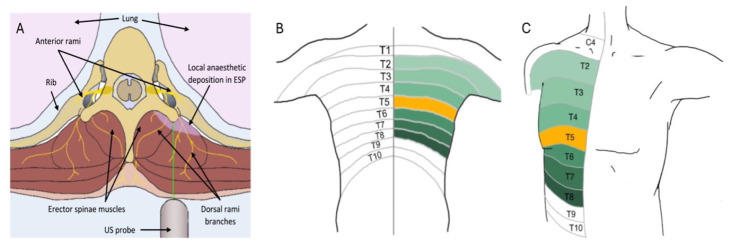
The Erector Spinae Plane (ESP) Block. (**A**) Axial cross section demonstrating US probe position and anatomical structures in ESP block. (**B**) Posterior dermatomes and expected spread (green) of ESP block at the level of T5 (yellow). (**C**) Anterior dermatomes and expected spread (green) of ESP block at the level of T5 (yellow).

Despite the lack of a precise in-depth understanding of the mechanism of some fascial plane blocks, evidence of their effectiveness in clinical practice has increased in recent years, as has their popularity [12,13,14]. Chest wall-specific blocks have been shown to be beneficial for patients in breast and thoracic surgery by minimising recovery times and reducing opioid requirements [15,16]. Foremost is the erector spinae (ESP) block which was first described in 2016 [17]. Its popularity continues to increase, largely owing to its relative technical ease and reassuring safety profile. It is a posterior chest wall block that targets the space between the erector spinae muscle sheath and the transverse process of vertebrae [18]. This results in analgesia from the back to the midline of the axilla. The extension of analgesia to the anterior chest may also occur but is unreliable [19]. The primary target of the LA agent is the dorsal rami of the spinal nerve, with extensions to the ventral rami and the intercostal and paravertebral spaces also seen [9,20,21]. As such, the ESP block is often compared with the paravertebral block (PVB), due to their similar target areas, but is associated with fewer complications [13,14]. ESP blocks (Figure 1) are also favoured for the wide anatomical area they can cover with a single injection resulting in effects at multiple vertebral levels: typically three in a cranial and three in a caudal direction [9,21,22,23,24]. For example, evidence shows that an ESP block performed at the level of T5 will result in analgesia from T3–T9 [17]. The block can be performed at all levels of the spine resulting in analgesia to a very wide variety of regions [19]. Another attractive characteristic of this block is its safety profile in the setting of anti-thrombotic drugs. As the ESP block is considered a superficial block, it is safe in patients undergoing such therapies, in contrast with deeper paravertebral or epidural blocks [25,26].

In two randomised controlled trials (RCTs), the ESP block was superior to the serratus anterior plane block for video-assisted thoracoscopic surgery (VATS), including reduced opioid consumption for up to 48 h postoperatively [27,28]. One used the patient-centric outcome quality of recovery-15 score (QoR-15), with ESP patients showing superior quality of recovery and a lower rate of postoperative complications as measured using the Comprehensive Complications Index [27]. The QoR-15 was also the primary outcome in an RCT investigating the effect of ESP block in thoracolumbar decompressive surgery, which showed that compared to no block, patients with bilateral ESP block had improved recovery and reduced pain up to 24 h postoperatively [29]. It was also compared with PVB in VATS, where ESP block was performed by an anaesthetist whilst the video-assisted PVB was performed by a surgeon. Both groups received an initial bolus followed by a continuous infusion of levobupivacaine over 48 h. The results showed a statistically significant improvement in the QoR-15 score at 24 and 48 h postoperatively in favour of the ESP block. No significant difference in opioid consumption was found [30]. A new question for the fascial plane block is whether LA delivered via a programmed intermittent bolus (PIB) regimen is better than via continuous infusion. PIB is well established as superior to continuous infusion in labour epidurals, for example [31]. Our group has just completed an RCT investigating continuous infusion versus intermittent bolus in the setting of ESP block in VATS. This showed the equivalence of QoR-15 and opioid consumption with either PIB or continuous infusion. While the PIB group had a marginally higher QoR-15 at 24 h postoperatively, it was not statistically significant (*p* = 0.29) [32].

With respect to breast surgery, meta-analyses have demonstrated that ESP block provides better analgesia, decreased pain scores, and statistically significant reduced opioid consumption at 24 h when compared with general anaesthesia alone [14]. The same analysis reported that when compared with PVB, patients with ESP blocks had high pain scores in the first 2 h postoperatively but at no other time point. Opioid consumption did not differ statistically. There was also a higher pooled incidence of pneumothorax in the PVB group (2.58% vs. 0% in the ESP group) [14]. When compared with intercoastal nerve block in thoracic surgery, a single-injection ESP block provided similar analgesia compared to a six-level ultrasound-guided intercostal nerve block with respect to opioid consumption and pain scores postoperatively [33]. Further studies in thoracic surgery are highlighted in a systematic review which identified 6 RCTs comparing ESP in thoracic surgery to either no block or PVB. This analysis showed that in ESP vs. no block, 24 h opioid consumption was reduced significantly in the ESP group but was equivalent when compared with PVB [16].

A number of limitations of facial plane blocks are outlined above including the unpredictability of spread and the anatomical variances that may result in patchy blocks. Circumventing these issues is a challenge that has not been explored extensively yet. It may be argued that current research regarding our understanding of the spread of LA in fascial plane blocks, and regional blocks in general is limited by the subjects in which much of these studies are performed, i.e., cadaveric patients. In these studies, variables such as intrathoracic pressure changes and tissue tension are not easily recreated [11]. Novel approaches to address these limitations are needed, and consensus on what constitutes a successful or failed block would improve consistency. Further clarification is also needed regarding the choice of equipment in fascial plane blocks and the standardisation of technique. Technical factors such as needle size, orientation, and injection pressure are largely underexplored. In addition, the influence of needle endpoint and injection speed has yet to be fully elucidated in these blocks [34]. Considering these are large-volume blocks, a stronger evidence base for optimal dose and concertation for fascial plane blocks would further add to their already promising safety profile [9].

Despite the number of unanswered questions that exist with respect to ESP blocks, there is a widespread appetite for it in the anaesthesia community [12]. With this wave of interest and positivity should also come a degree of caution with respect to publication bias. One narrative review reported that when investigating the clinical uses for ESP block, of 23 RCTs, only 7 were high quality. Weaknesses in the remaining 16 RCTs were attributed to faults such as discrepancies in protocols and errors in registered versus reported protocols [35]. Caution should always be employed without evidence that any new block is at least equivalent or superior to our existing methods [36].

## 3. Neuromodulation Techniques for Acute Pain

While the concept of neuromodulation is well established in the field of chronic pain, recent advances in the technology are being investigated for its application in the management of acute pain [37]. Neuromodulation is defined as the “modification of neurological function, including both neuronal and glial cell activity, through delivery of an electrical, magnetic or chemical stimulus, to specific neurological targets” [38]. There are many different approaches to neuromodulation including spinal cord stimulation, transcutaneous electrical nerve stimulation (TENS), and peripheral nerve stimulation (PNS) [39,40]. PNS is a method that has gained increasing attention in the field of regional anaesthesia for its application in managing acute postoperative pain [37,41,42,43,44,45]. PNS involves the implantation of electrodes in the close vicinity of a target nerve. An external pulse generator is responsible for the delivery of electrical pulses through the implanted electrode [46]. The last decade has seen the development of minimally invasive percutaneous peripheral nerve stimulation (pPNS), which consists of the ultrasound-guided percutaneous implantation of small (0.2 mm) monopolar, coiled electrical leads [47].

**Figure 2:**
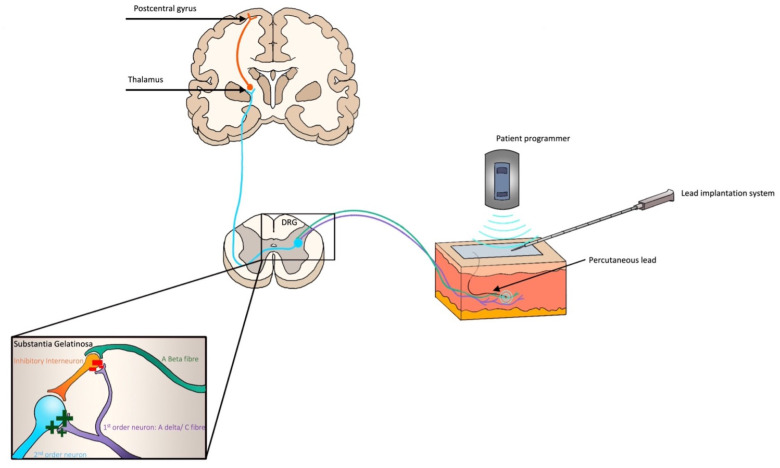
Neuromodulation via percutaneous peripheral nerve stimulation for acute pain management. Stimulation of A Beta fibres peripherally increases the action of the inhibitory interneuron, thus reducing the transmission of pain signals from A Delta and/or C fibres onto the second (blue)- and third (orange)-order neurons.

The mechanism by which PNS is understood to work (Figure 2) is largely grounded in the Gate Theory of pain [48,49]. More recently, both alternative and complementary mechanisms have been proposed, including theories at both peripheral and central levels [46,50,51]. At a peripheral level, PNS acts to reduce local inflammatory mediators and blood flow. It has also been shown to downregulate inflammatory neurotransmitters and endorphins, while electrophysiological studies have shown it reduces the transmission of efferent nociception [50,52]. While these mechanisms likely contribute to analgesia, the dominant mechanism of action is believed to be through the stimulation of Aβ fibres. When activated, these inhibit pain transmission at the dorsal root ganglion (DRG) between the first- and second-order neurons via an inhibitory interneuron in the substantia gelatinosa of the spinal cord [53].

In 2018, the Food and Drug Administration approved the use of pPNS for use in acute postoperative pain [41]. It has potentially beneficial qualities with respect to acute pain management including opioid sparing and the absence of sensory, motor, or proprioceptive deficits which may benefit patient rehabilitation [42,43]. The risk of infection is <1 per 32,000 indwelling days, and the leads are approved for use for up to 60 days [54]. Lead placement is typically 1–2 cm away from the target nerve which may reduce the risk of neurological injury [45]. The leads are also a potential limitation, however, because they can fracture or dislodge and may be left in situ [44]. Other questions such as the optimal distance between the lead and target nerve, the implications of tissue impendence, the consistency of electrical current, the and long-term effects of prolonged use remain unanswered [55,56].

A role for pPNS in ambulatory orthopaedic surgery has been proposed in a pilot randomised sham-controlled trial. Preoperatively, a lead was placed percutaneously to target the sciatic nerve for major foot or ankle surgery or anterior cruciate ligament repair. The brachial plexus was targeted for patients undergoing rotator cuff repair. Postoperatively, patients were randomised to a sham or electrical stimulation group via an external pulse generator in a double-blinded approach [45]. The authors concluded that pPNS led to a statistically significant improvement in analgesia and reduced opioid requirement, which lasted for 7 days postoperatively. Of note, all patients in this study received a single-injection peripheral nerve block immediately after the lead implantation and before the start of surgery [45]. Some individuals from the same research team had previously published their findings regarding the use of peripheral nerve stimulators for rotator cuff repair. In this study, patients were randomised to either a stimulation or sham group and did not receive any peripheral nerve block. Eleven of a total of sixteen patients, however, required a rescue block prior to discharge and overall no analgesic effect immediately postoperatively was appreciated [42]. These were small studies limited by a number of technical challenges. Overall, superiority over current methods cannot be inferred from the current research and future comparative studies are warranted.

## 4. Pharmacological Advances

The pursuit of the pharmacological agent(s) that will result in the “ideal block” continues. With such an agent, one could theoretically prolong a good-quality block duration in a predictable manner without side effects for patients [57]. Duration is one of the greatest limitations of regional anaesthesia and traditional one-injection blocks last for a maximum of 8–14 h [58]. Increasing efforts are being made to address this through both pharmacological and non-pharmacological approaches in the form of catheters [59]. Three main pharmacological avenues have been explored with respect to block prolongation, namely intravenous adjuncts, perineural adjuncts, and sustained-release LA molecules.

Sustained-release LA molecules constitute the most recent advance in pharmacology for regional anaesthesia [60]. Liposomal bupivacaine was approved by the Food and Drug Administration in 2011 for surgical site infiltration. In November 2023, two further indications were approved—adductor canal and sciatic nerve block [61]. The availability of this agent is currently limited and it is costly [62]. Initial studies on liposomal bupivacaine were in the context of local infiltration [63]. Liposomal bupivacaine was compared with bupivacaine hydrochloride in interscalene block where “modest” effects in favour of the liposomal agent were reported in the highest pain scores during the first week postoperatively [64]. A meta-analysis investigating liposomal versus non-liposomal bupivacaine for peripheral nerve blockade encompassing nine trials had a primary outcome of the difference in rest pain score 24–72 h post-blockade. Liposomal bupivacaine did not meet the predefined threshold for clinical significance. For all other secondary outcomes, liposomal bupivacaine was similar to non-liposomal [65]. Therefore, there seems to be no benefit of liposomal agents over current drugs, and this combined with their high cost makes them unlikely to be adopted in the near future.

## 5. Regional Anaesthesia outside the Operating Theatre

The expansion in regional anaesthesia techniques in recent years is appreciated both inside and outside the surgical theatre environment. Regional anaesthesia has become well established outside of the theatre environment, both in the emergency department and through the establishment of acute pain services. In emergency departments, regional techniques can be used for the management of pain relating to trauma such as rib and hip fractures. This has been shown to have positive effects with respect to opioid use, patient satisfaction, and hospital length of stay [24,66,67]. An acute pain service is an integral function in large institutions dealing with major surgery and trauma [68]. The administration of opioids has long been the mainstay analgesic strategy for acute trauma; however, the inappropriate and prolonged use of opioids may be an important factor implicated in the current opioid epidemic [69,70,71]. An acute pain service plays a critical role in ensuring appropriate opioid prescribing stewardship and early regional anaesthesia interventions which provide immediate, short and long-term benefits [3].

An acute pain service is not limited to surgical patients and may also manage injuries that warrant conservative management. Equally, these injuries may be managed earlier in a patient’s presentation by non–anaesthetists in the emergency department [24]. Examples of such injuries include, but are not limited to rib fractures, pubic rami fractures, clavicle fractures, and soft tissue injuries. Rib fractures are associated with increased morbidity and mortality, especially among the elderly. In the majority of cases, treatment is conservative and satisfactory analgesia is paramount to prevent associated respiratory complications [72]. For some time, thoracic epidural has been considered the “gold-standard” regional anaesthesia technique for managing severe pain in this patient cohort [73]. One of the useful features it offers is bilateral analgesia after a single procedure [74]. However, its use is limited by a number of contraindications such as coagulopathy and the presence of paraspinal infection [26,75]. However, in comparison to a thoracic epidural, ESP block is a fascial plane block with a needle injection site far away from the epidural space. Therefore, the risk of a devastating neuraxial haematoma following the insertion of an ESP catheter in the presence of coagulopathy is very rare and, thus, offers a more favourable safety profile [26]. Early retrospective studies highlighted that ESP block improves pulmonary function, pain scores, and opioid requirements in trauma patients [76,77]. ESP catheter use has been well received by patients, with one small quality assurance initiative report involving 29 patients highlighting that patient satisfaction was dramatically improved after the placement of an ESP catheter [78].

A small RCT (n = 50 patients) demonstrated that continuous ESP block was equivalent to thoracic epidural analgesia for thoracic trauma with respect to a number of parameters including analgesic effect and pulmonary function. The only statistically significant difference between the two interventions was a lower recorded mean arterial pressure in the epidural group (*p* < 0.001) [79]. Incorporating a continuous ESP catheter as part of a multimodal analgesia appears to be a promising intervention in thoracic trauma patients but at present, there is a lack of high-quality level 1 evidence to support this. A large multicentre RCT (ESPEAR TRIAL) is currently underway, comparing the continuous ESP catheter technique plus multimodal analgesia versus sham ESP catheter plus multimodal analgesia [80]. Results from this trial should further guide the role of regional anaesthesia in thoracic trauma.

Another example of an orthopaedic traumatic injury, often managed conservatively, is pubic rami fractures. This type of pelvic injury is commonly encountered in patients who are female, 80 years of age or older, and have a history of osteoporosis; and it may occur in low- or high-energy traumas [81,82]. Pain associated with this type of injury can be very severe and limits mobilisation. Regional anaesthesia for this injury is challenging because of the desired goal of adequate analgesia while avoiding inadvertent motor block. With this in mind, the pericapsular nerve group (PENG) block may possibly have a role. The PENG block is a novel fascial plane block recently described and utilised as an analgesic option for traumatic hip fractures and for elective hip arthroplasty [83,84]. This nerve block targets the articular branches of the femoral, obturator, and accessory obturator nerves to the hip capsule while sparing the motor branches [85]. A recent case report described the successful implementation of this block by an acute pain service for a patient with traumatic superior and inferior public rami fractures, allowing the patient to mobilise [86]. This finding is echoed in another case series of PENG blocks for pelvic bone fractures, involving in one case an inferior pubic rami fracture and in another case both superior and inferior public rami fractures. Both cases in this series were regarding blocks carried out by emergency medicine physicians [87]. Further studies examining the PENG block for this indication are warranted. However, this technique requires rigorous evaluation in an RCT comparing its efficacy and safety with an established regional technique such as ilioinguinal and iliohypogastric blocks combined.

## 6. Rebound Pain after Regional Anaesthesia

A significant challenge that is largely unaddressed in regional anaesthesia is rebound pain. Rebound pain is a transient acute, clinically significant pain that arises upon the regression of a peripheral nerve block [88]. Its incidence is unclear, but given the increase in regional anaesthesia use, it is likely to be common. The phenomenon of rebound pain is not fully understood but is associated with single-injection peripheral nerve blocks, and frequently occurs at night, which may be explained by a daytime block regressing after 6–12 h [89]. A question regarding rebound pain remains unanswered—is it due to the re-emergence of previously blocked surgical pain, or is it an exaggerated nociceptive response somehow caused by regional anaesthesia? Patient factors, systemic inflammation, brain cortical processing, surgical factors, and regional anaesthetic techniques have all been hypothesised as explanations [90].

Continuous catheter techniques may mitigate rebound pain by prolonging block effects long enough to allow for more healing, reduced inflammation, and a less sudden offset of analgesia [90,91]. A randomised control trial of patients (n = 71) undergoing rotator cuff repair had three arms: general anaesthesia only, single regional interscalene injection, or continuous catheter interscalene block. The general anaesthesia-only patients received a standardised general anaesthetic. The single-injection block group received 20 mL of 0.5% ropivacaine through a needle. The continuous catheter group received the same initial 20 mL block through a catheter followed by a continuous infusion of 0.2% ropivacaine at 5 mL/h with a patient-controlled bolus of 5 mL hourly for 48 h. The data were collected on postoperative days 1, 2, 3, and 7. Severe pain, i.e., 8–10 on a numerical rating scale (NRS) on day 1 postoperatively was reported in 40%, 78%, and 15% in these respective groups. On day 2 postoperatively, the trend in favour of the continuous catheter continued with just 10% reporting severe pain compared to 35% in the other two groups. By the end of the seven days, just 26% of patients with a continuous catheter had reported NRS ≥4 compared to 58% in the general anaesthesia group, and 83% in the single-injection group (*p* ≤ 0.05) [92]. Continuous catheter techniques, however, are technically challenging, are associated with complications such as failure and infection, and are labour-intensive to manage making them less accessible in some circumstances [93].

Efforts to optimise catheter techniques are ongoing. Until recently, the most common technique of using regional anaesthesia catheters was by continuous infusion or patient-controlled boluses. Whether programmed intermittent bolusing is advantageous remains uninvestigated [94]. Nuanced approaches such as using start-delay timers have been proposed for their ability to prolong the duration of analgesia [95]. A retrospective study among patients undergoing wrist surgery receiving a continuous infraclavicular block suggested that after an initial bolus, the delayed onset of LA infusion maximised LA availability and prolonged the block. This proposal warrants an RCT [96].

## 7. Future Directions

The interest that regional anaesthesia has generated amongst anaesthetists is reflected in the growing memberships of societies such as the European Society of Regional Anaesthesia (ESRA) [97,98]. As the weight of supporting evidence for the use of regional anaesthesia has grown in recent years, so too has the emphasis on its integration into core teaching as part of the anaesthesia training systems both in the UK and Ireland [99,100]. While regional anaesthesia in the past was often limited to enthusiasts, it is now evolving into an expected component of a trained anaesthetist’s skillset. The UK’s “plan A blocks” concept focuses training on a core set of well-established blocks that serve as a basic skillset for the trainee anaesthetist to improve patient outcomes [36]. Given its relative lack of technical complexity, the ESP block is one of the seven such plan A blocks, and yet, when surveyed, trainee anaesthetists reported that only 10% of them felt confident about performing an ESP block with remote supervision compared to 60% for axillary blocks [101]. Future anaesthesiology training programmes should aim to deliver competency in the basic skills of ultrasound, needling technique, and core blocks.

Given the rate at which artificial intelligence is developing across all systems across the globe, its role in regional anaesthesia will likely increase in the future. Indeed, a number of recent publications have already looked at the role of such technologies in regional anaesthesia education and training [102,103]. Image interpretation is essential for successful and safe regional anaesthesia. Assistive technology has the potential to aid in defining structures and identifying targets using colour overlay. This is something that has briefly been explored in small studies thus far [103,104]. Future technology may enhance psychomotor competencies such as needle visualisation, image optimisation, image interpretation, and mapping the spread of LA. Just as ultrasound led to increased uptake and improved outcomes in regional anaesthesia, new technology might potentially add further improvement. Quality control and rigorous RCTs should remain the priority for regional anaesthesia developments [104].
